# Comparison of the Jcerity Endoscoper Airway with the LMA supreme for airway management in patients undergoing cerebral aneurysm embolization: a randomized controlled non-inferiority trial

**DOI:** 10.1186/s12871-022-01666-w

**Published:** 2022-04-26

**Authors:** Junfei Zhou, Lu Li, Fang Wang, Yunqi Lv

**Affiliations:** 1 Department of Anesthesiology, Pain and Perioperative Medicine, The First Affiliated Hospital of Zhengzhou University, Zhengzhou, 450052 China; 2 Department of Pain Medicine, the Second Affiliated Hospital of Zhengzhou University, Zhengzhou, China

**Keywords:** Laryngeal mask airway, Airway management, Cerebral aneurysm embolization, Anesthesiology

## Abstract

**Background:**

Jcerity Endoscoper Airway is a new back-open endoscopic laryngeal mask airway device with a unique design. Our study sought to compare the implantation, ventilation quality and complications of JEA (Jcerity Endoscoper airway) versus LMA (Laryngeal Mask Airway) Supreme in the procedure of cerebral aneurysm embolization.

**Methods:**

In this prospective, randomised clinical trial, 182 adult patients with American Society of Anesthesiologists class Ι-II scheduled for interventional embolization of cerebral aneurysms were randomly allocated into the Jcerity Endoscoper airway group and the LMA Supreme group. We compared success rate of LMA implantation, ventilation quality, airway sealing pressure, peak airway pressure, degree of blood staining, postoperative oral hemorrhage, sore throat and other complications between the groups.

**Results:**

There were no significant differences between the groups in terms of one-time success rate of LMA implantation, ventilation quality, airway sealing pressure or airway peak pressure. However, LMA Supreme group showed a higher degree of blood staining than the JEA group when the laryngeal mask airway was removed (*P* = 0.04), and there were also more oral hemorrhages and pharyngeal pain than JEA group (*P* = 0.03, *P* = 0.02). No differences were observed between groups in terms of other airway complications related to the LMA.

**Conclusions:**

The JEA could not only achieve comparable one-time success rate of implantation and quality of ventilation as the LMA Supreme, but also have lower blood staining degree of mask and less sore throat in patients undergoing perioperative anticoagulation for cerebral aneurysm interventional embolization.

**Trial registration:**

Chinese Clinical Trial Registry, ChiCTR2100044133; Registered 11/03/2021.

**Statement:** This study adheres to CONSORT guidelines.

## Introduction

With the popularization of endovascular techniques, interventional embolization of unruptured cerebral aneurysms with general anesthesia under digital subtraction angiography (DSA) has become the primary treatment [1–6]. In order to avoid aneurysm rupture and bleeding, the maintenance of hemodynamic stability and fully sedated during the perioperative period is important factor that should be taken into account [7, 8].

However, patients undergoing cerebral aneurysm embolization generally need anticoagulation and antiplatelet therapy before surgery [9, 10], and anticoagulation treatment during and after surgery. This anticoagulation treatment would become a challenge for the airway management during the anesthesia. During the procedure of cerebral aneurysm embolization with LMA Supreme (Teleflex Medical, Athlone, Co. Westmeath, Ireland), we found that patients who received anticoagulant and antiplatelet aggregation therapy experienced increased pharyngeal pain and bleeding after surgery. Therefore, based on the characteristics of this kind of surgery for perioperative anticoagulant patients, we need a ventilation device that not only ensures effective ventilation but also reduces oropharyngeal injuries.

**Fig. 1:**
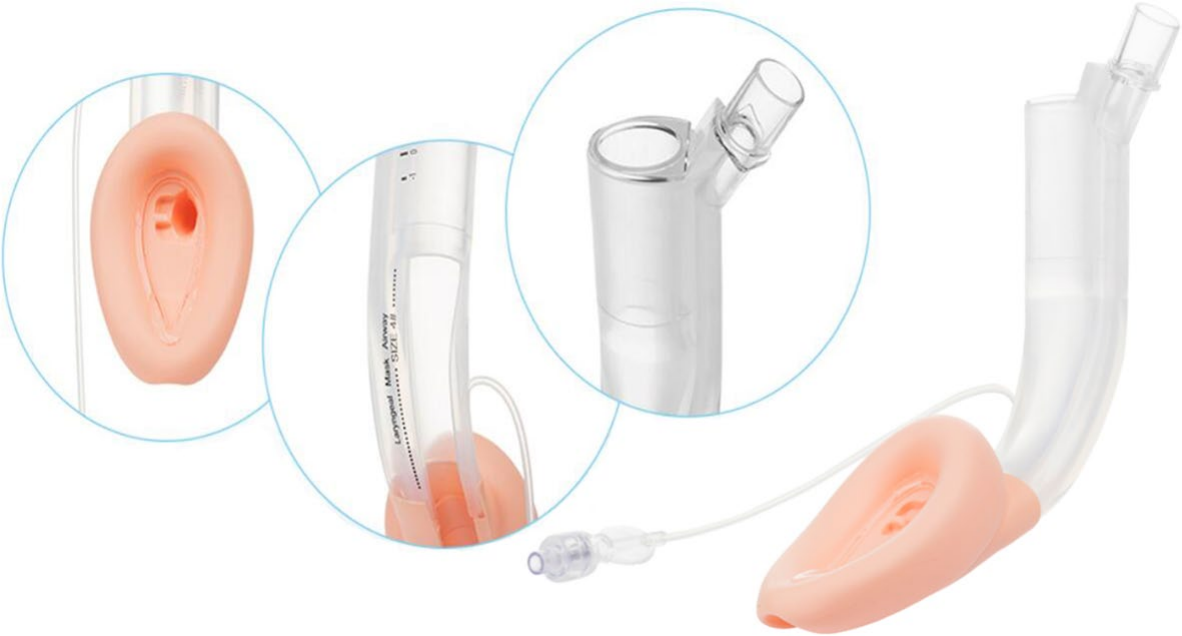
The design features of JEA,which were shot and edited by Junfei Zhou

The JEA (Zhejiang Jcerity Medical Technology Co., Ltd., Huzhou, China) was originally designed for painless gastroscopy and treatment (Fig. 1). As a new type of LMA specially developed for upper gastrointestinal endoscopy, JEA’s innovation is to add a dedicated endoscopic channel (20*22 mm inner diameter) that runs in parallel with an independent airway channel with a terminal cuff. Its endoscopic examination channel is semi-enclosed, which greatly reduces the friction area between the laryngeal mask and the posterior pharynx wall during the insertion process. Our hypothesis was that the differences in shape and materials of the JEA and the LMA Supreme would result in differences in airway management in patients receiving perioperative anticoagulation.

Therefore, the main purpose of this study was to compare the implantation, ventilation quality and complications associated with JEA and LMA Supreme in aneurysm embolization, so as to further optimize the anesthesia airway management mode of such operations.

## Materials and methods

**Fig. 2:**
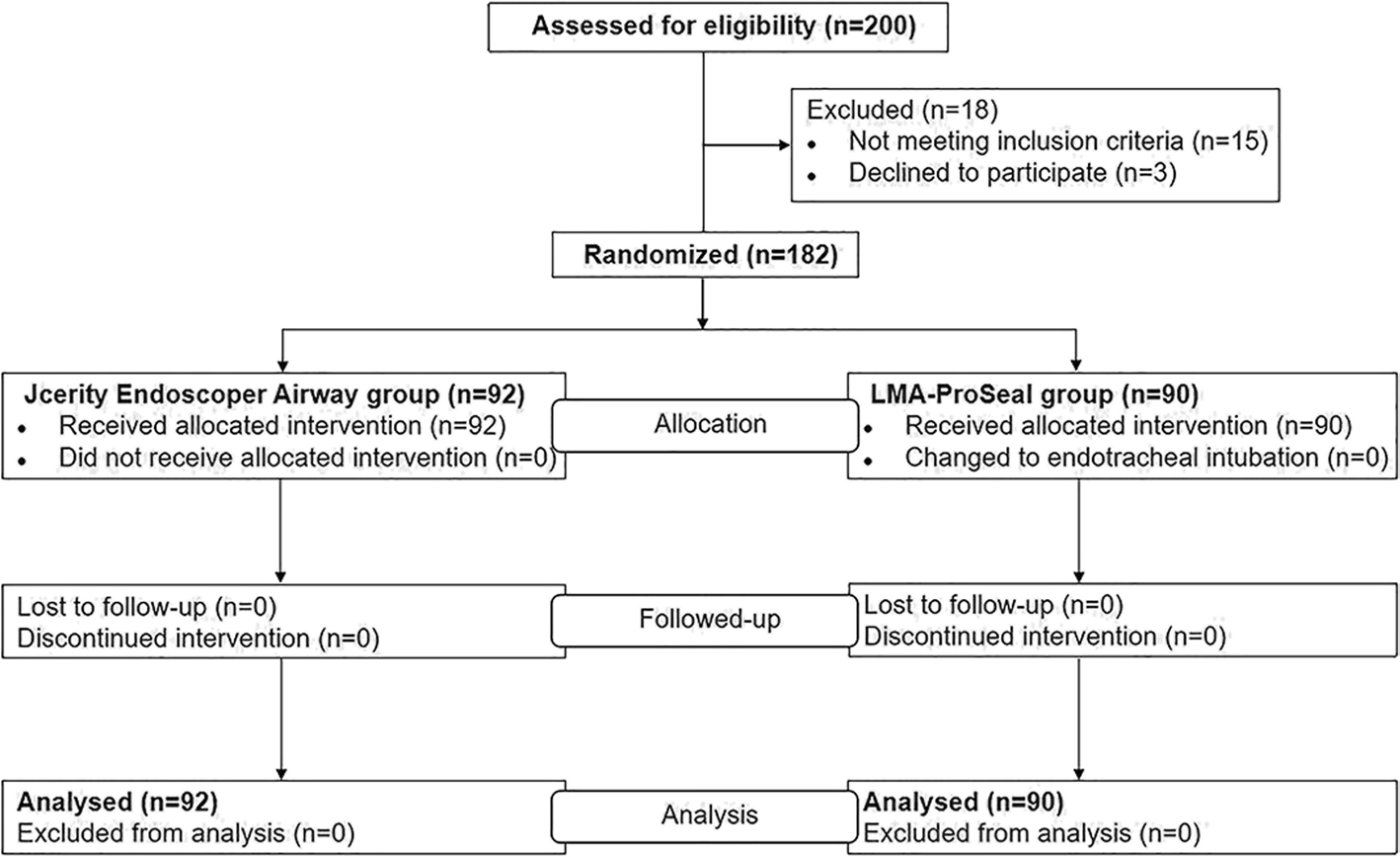
Patient enrolment and flow

We conducted a randomized, controlled, non-inferiority trial between April 2021 and July 2021 in the First Affiliated Hospital of Zhengzhou University in China. This trial was approved by the ethics committee of the First Affiliated Hospital of Zhengzhou University (2019-KY-276), and each patient provided informed written consent. All methods were carried out in accordance with relevant guidelines and regulations and with CONSORT recommendations. Prior to patient enrollment, the trial was registered as a clinical trial at Chinese Clinical Trial Registry (Registration No. ChiCTR2100044133, Date of first registration: 11/03/2021). The first patient was recruited and registered on April 1, 2021.We studied 200 adult patients with physical status of I-II according to the American Society of Anesthesiologists, who were scheduled for elective cerebral aneurysm embolization under general anesthesia. Exclusion criteria were as follows: neck deformity; high risk of aspirating stomach contents; known or expected difficult airways; obesity (body mass index [BMI] > 30 kg.m^−2^; estimated operation time > 3 h; reduced lung compliance; incisor defect; mouth opening limitations. Patients were divided to JEA group and LMA Supreme group. A biostatistician independent of data management conducted the randomization using SAS 9.4 software (SAS Institute,Cary,NC) to generate random numbers (with a ratio of 1:1). Therefore, the final study population included 182 patients who were randomly divided into two groups: the JEA group (*n* = 92) and the LMA Supreme group (*n* = 90). The study flow diagram is showen in Fig. 2. The LMA size was selected according to the patient’s weight. The investigators, each with more than five years of anesthetic experience, were proficient with two airway devices, each having performed more than 100 LMA Supreme and 100 JEA placement experiences in anesthetized patients prior to the start of the trial. Before anesthesia, the anesthesiologist in charge of the patient got the allocation information from the researcher conducted the randomization. The same method of anesthetics was employed for all patients. During the operation, patients were not aware of which type of LMA was used. For practical reasons, investigators in charged of recording management details were not blinded but responsible for the information of postoperative complications remained blinded to the LMA. Data Analysts were also blinded to the LMA .

Thirty minutes before anesthesia, all patients were given penehyclidine hydrochloride 0.01 mg·kg by intravenous injection. All patients were routinely monitored using electrocardiogram, pulse oximetry (SpO_2_), non-invasive blood pressure measurement, and determination of end-tidal carbon dioxide and bispectral index (BIS). Before induction, patients were pre-oxygenated for no less than 3 min. Inducing drugs included etomidate (0.2 To 0.3 mg•kg^−1^), sufentanil (0.1ug·kg^−1^), cisatracurium (0.15 to 0.2 mg•kg^−1^) and dezocine 0.1 mg•kg^−1^. The selected JEA or LMA Supreme was inserted according to the manufacturer’s instruction manual. Before insertion, each LMA was lubricated with Obucaine gel. Volume controlled ventilation (tidal volume 6–8 ml·kg^−1^) was used to verify the size and position of the LMA device. Optimal ventilation defined as normal thorax expansion, normal airway pressure without air leak and normal pressure-volume curve [11]. If the lungs could not be ventilated, another insertion attempt was made, during which time the jaw was allowed to be lifted. If the patient still couldn’t be ventilated, the device changed to another size. If there was still no ventilation, the LMA was abandoned and replaced with endotracheal intubation. After the LMA was placed, agastric tube was inserted 40 to 55 cm to aspirate gastric contents. If the gastric tube couldn’t be inserted, the patient was excluded from the study. Once ventilation was successful and the gastric tube was easily inserted, the LMA was secured with tape. Cuff pressure was monitored using a handheld manometer (Ambu, Ballerup, Denmark) to achieve 60 cmH2O in both devices [12].

Mechanical ventilation was performed at tidal volumes of 6–8 ml·kg^−1^ and a certain frequency, and the end-expiratory CO_2_ was maintained in the normal range. End-expiratory carbon dioxide and pressure volume curves were recorded throughout the procedure.

During the operation, continuous infusion of sevoflurane and remifentanil was used to maintain the BIS between 40 and 60. According to the length of the operation, cisatracurium was added to prevent head movement that was caused by respiratory confrontation due to the recovery of spontaneous breathing (head micromotion often produced artifacts under DSA and affected the operation). The number of LMA attempts with or without assistance was recorded by an independent observer, and the success rate of the first or second placement was recorded. We recorded the duration of implantation time.

We recorded complications related to airway management: tooth or mucosal trauma; cough; hiccup; laryngospasm; bronchospasm; high inspiratory pressure (>25 cm H_2_O); SpO2 < 95%; and reflux. When hiccup, laryngospasm, bronchospasm, leakage or high inspiratory pressure occurred, we increased the depth of anesthesia or administered neuromuscular blockers. If high inspiratory pressure or audible leakage was detected, we adjusted the position of the LMA or the patient’s head.

After one hour in the recovery room, a structured interview was conducted to investigate the patient’s sore throat, which was recorded by ablinded observer. The severity of sore throat was graded according to three grades: mild, moderate, or severe. Three hours after the operation, a blinded observer was admitted to the ward to follow up complications related to LMA placement (postoperative oral bleeding, maxillary hematoma, and vocal cord paralysis).

### Outcomes and definition

The primary endpoint was one-time success rate of implantation which defined as a successful insertion on first attempt after induction of anaesthesia. The secondary endpoints included quality of ventilation and complication related to airway management. Quality of ventilation was scored on a 3-point scale [11]: (1) Optimal ventilation was defined as normal thoracic expansion, normal pressure-volume curve and square wave diagram without air leakage; (2) Ventilation difficulty was defined as peak airway pressure > 25 cm H2O or severe air leakage related to mechanical ventilation failure; (3) After all attempts to ventilate failed, the case was changed to endotracheal intubation. If ventilation difficulty was managed by adjusting the insertion depth, changing the head and neck position or adjusting the cuff volume, the score was recorded as 2 points. Complication included blood staining degree of the mask, sore throat, postoperative oral bleeding and other airway injury. The degree of blood staining was recorded after the laryngeal mask was removed. The criteria for judging the degree of blood staining of LMA were as follows: if a small amount of blood was detected on the pulled LMA, it was recorded as “mild”; if the blood was limited to a quarter of the LMA surface, it was classified as “moderate”; if the blood covers at least half of the LMA surface, it is classified as “severe”. In case of moderate or above blood staining, the oral mucosal damage was observed using laryngoscopy 6 h after operation.

Additional prespecified endpoints included time taken of implantation (from the time when laryngeal mask started to be inserted until laryngeal mask was fixed with adhesive tape), the number of attempts to insert, peak airway pressure, airway sealing pressure and fiber optic bronchoscope (FBS) view of vocal cords (VC). Peak airway pressure was recorded at the beginning of controlled ventilation; Airway sealing pressure [11] was measured by closing the expiratory valve and observing the balanced airway pressure under the fresh air flow of 3 L·min^−1^. When the peak airway pressure did not rise or there was air leakage in the mouth, the airway pressure was the maximum leakage pressure. If the maximum air leakage pressure was less than 15 cm H_2_O, the LMA was replaced. If there was gas leakage in the mouth, we opened the expiratory valve to avoid alveolar trauma. If the maximum pressure reached 40 cm H_2_O, we recorded 40 cm H_2_O as the airway sealing pressure. Airway sealing pressure was recorded at various time points (T1: immediately after LMA implantation; T2: 15 min after T1; T3: 30 min after T1; and T4: at the end of the operation); FBS view of VC: When LMA was successfully inserted, a 4.0-mm FBS(F1-10RBS, Pentax, Japan) was introduced near the end of laryngeal mask through it’s airway channel by the same anesthesiologist familiar with FBS. If resistance was encountered, operated the tip of FBS and classified the laryngoscope view. The position of the laryngeal mask was determined by FBS. The optimal positioning was defined as that the tip of laryngeal mask was located behind the arytenoid cartilage, epiglottis was not folded or blocked the airway, and the vocal cords could be seen. Any deviation from these standards was considered to be suboptimal positioning.

### Statistical analysis

The required sample size to show non-inferiority of JEA versus LMA Supreme based on the primary outcome measure of one-time implantation rate was estimated to be 176 patients. This estimation was calculated on the basis of an expected one-time implantation rate of 93% for JEA and 96.7% for LMA Supreme [11] with non-inferiority established if the upper limit of the two-sided 95% CI% of the absolute risk difference was less than 12% (non-inferiority margin) and the sample size was set to ensure at least 80% power (1–β = 0.8) at a significance level of α = 5%. Considering a 5% missed follow-up rate, we collected 92 effective cases in JEA group and 90 cases in LMA Supreme group finally. Statistical analysis was performed using Statistical Package for Social Sciences (SPSS Inc., version 24.0 for Windows, Chicago, IL, USA). Continuous variables were evaluated for normality using the Kolmogorov–Smirnov statistic (*P* > 0.05 indicated normality). Normally distributed continuous variables were presented as mean ± standard deviation (SD). An unpaired t-test was used to compare continuous variables, the Mann-Whitney test was used to compare skewed variables and the χ2 test or Fischer exact test was used to compare categorical variables between Jcerity Endoscoper and LMA Supreme groups. All statistical tests were two-sided and *p* values <0.05 were considered significant.

## Results

No differences were observed between the two groups in terms of age, sex, BMI or duration of surgery (Table 1).

**Table 1:** Patient characteristics and surgical data

Patients	JEA (*n* = 92)	LMA Supreme (*n* = 90)	*P*
Age (y)	57.78 ± 1.24	55.78 ± 1.29	0.75
Sex (F/M)	30/62	31/59	0.88
BMI	23.91 ± 0.32	24.33 ± 0.29	0.26
Duration of surgery(min)	105.50 ± 3.42	101.70 ± 3.70	0.52

Data are expressed as number of patients or mean ± SD or absolute numbers

*BMI* body mass index, *JEA* Jcerity Endoscoper Airway, *LMA* laryngeal mask airway

All patients successfully completed the operation and returned to the ward safely. No adverse events of ventilation occurred during the operation. Except for one patient in LMA Supreme group who needed to be changed to endotracheal intubation, all patients successfully underwent LMA. The success rate of one-time implantation in JEA group was 93.48%, and that of the LMA Supreme group was 96.67%. There was no significant difference between the two groups. There were five patients in the JEA group and two in the LMA Supreme group who required a second attempt to implant by evacuating the gas in the cuff or lifting the mandible. One patient in JEA group was successfully ventilated by changing LMA to one size smaller, and there was no significant difference between the two groups. No differences were observed between the two groups in terms of LMA insertion times, number of insertion attempts, quality of ventilation, peak airway pressure, or FBS view of VC (vocal cords) (Table 2).

**Table 2:** Airway management details

Management Details	JEA (*n* = 92)	LMA Supreme (*n* = 90)	*P*
Implantation			
Time (s):	20.64 ± 0.40	20.62 ± 0.35	0.25
No. attempts(1/2/3):	86/5/1	87/2/1	0.53
one-time success rate:	93.48%	96.67%	0.50
Quality of ventilation (1/2/3)	89/3/0	88/2/1	0.55
Peak airway pressure(cm H_2_O)	16.87 ± 0.27	17.02 ± 0.25	0.68
FBS view of VC (optimal/suboptimal)	87/5	86/4	0.76

Data are expressed as number of patients or mean ± SD

*FBS* fiberscope, *JEA* Jcerity Endoscoper Airway, *LMA* laryngeal mask airway, *VC* vocal cords

*P ≤ 0.05 (JEA vs.LMA Supreme)

**Fig. 3:**
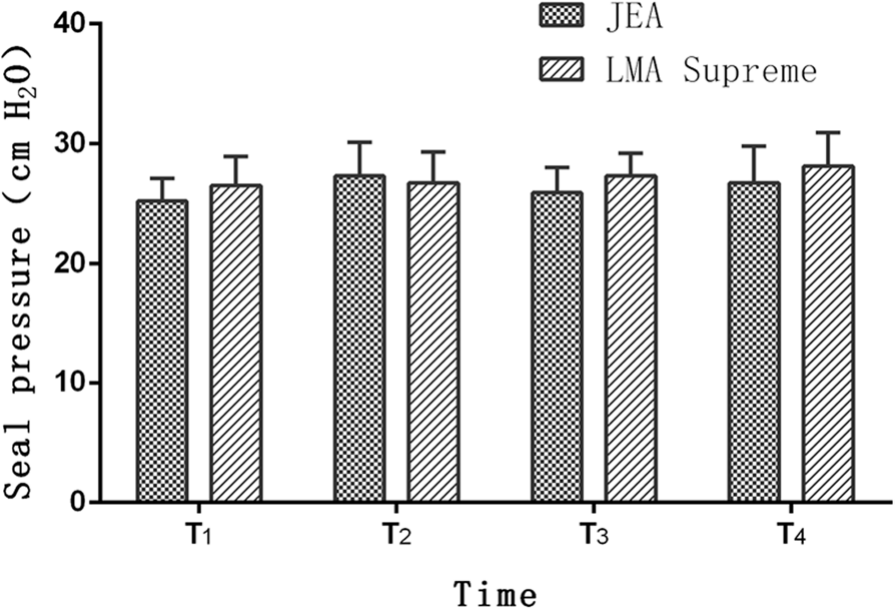
Seal pressure of JEA and LMA Supreme groups at various points. Data are represented as number of patients or mean ± SD. There was no difference between the two groups. *P ≤ 0.05 (JEA vs. LMA Supreme)

Compared with LMA Supreme groups, the sealing pressures of the airway at T1–T4 in the JEA group were 27.82 ± 4.15, 27.34 ± 3.89, 26.25 ± 3.02, 25.12 ± 3.01, respectively. There was no significant difference between the two groups (*P* > 0.05) (Fig. 3).

In patients receiving perioperative anticoagulation, operations on the airway can easily cause various injuries. In the LMA Supreme group, there were five patients with oral bleeding after returning to the ward, and one patient was estimated to bleed more than 200 ml. Based on visual laryngoscopy, the causes of bleeding were as follows: two were oral mucosal injuries, two were maxillary hematomas, and one was a lingual frenulum injury. In contrast, there was no significant oral bleeding in the JEA group. Only one patient felt a sore throat, and visual laryngoscopy after surgery revealed a tongue frenulum injury. Compared with the LMA Supreme group, the degrees of mask body blood staining and sore throat were significantly lower in the JEA group (*P* = 0.04 and *P* = 0.03). Complications such as laryngeal nerve injury, vocal cord paralysis, and arytenoid cartilage dislocation were not observed in either group (Table 3).

**Table 3:** Postoperative complications related to laryngeal mask placement

Associated complications	JEA (*n* = 92)	LMA Supreme (*n* = 90)	*P*
Blood staining degree of the mask(mild/moderate/sever)	3/1/0(4%)	12/3/1(17%)	0.04*
Sore throat (mild/moderate/sever)	6/1/0(7%)	17/3/1(23%)	0.03*
Postoperative oral bleeding	0	5(5%)	0.02*
Tongue frenulum injury	1(1%)	2(2%)	0.55
Laryngeal nerve injury or vocal cord paralysis	0	0	
Arytenoid cartilage dislocation	0	0	
Maxillary hematoma	0	2	0.15
Difficulty swallowing	3(3%)	7(7%)	0.18
Lingual nerve palsy	0	0	

Data are represented as number of patients or mean ± SD

*JEA* Jcerity Endoscoper Airway, *LMA* laryngeal mask airway

*P ≤ 0.05 (JEA vs. LMA Supreme)

## Discussion

In our study, it was observed that there were no significant differences between two groups in terms of one-time success rate of LMA implantation. We did not find any significant differences between two groups with respect to ventilation quality and airway sealing pressure. However, based on the results of postoperative complications, the JEA had lower degree of mask body blood staining, less sore throat and oral bleeding than LMA Supreme.

As a supraglottic ventilation tool, LMA avoids direct irritation to glottis and trachea. It has been widely used since its introduction in the US in 1988 [13–15]. The double lumen LMA is used in various operations because it greatly reduces reflux and aspiration [16]. Previous study showed that anesthesia using LMA (Laryngeal Mask Airway) during general anesthesia reduced hemodynamic fluctuation during surgery compared to endotracheal intubation [17, 18].

The most important aspect that should be considered is the ease of insertion. Several clinical studies have compared the first success rate of implantation in LMA Supreme and LMA Proseal with different results [11, 12, 19]. In our study,we found although one-time success rate of implantation (93.48%) in JEA group seemed to be lower than that in LMA Supreme group (96.67%), there was no statistical difference(*P* = 0.5), as same as insertion time and number of insertion attempt (Table 2), which showed that the implantation of JEA had the same convenience as LMA Supreme.

There were no significant differences in terms of quality of ventilation, peak airway pressure, FBS view of VC (Table 2) or sealing pressure (Fig. 3), which confirmed that JEA could offer anesthesiologist a credible alternative to LMA Supreme in terms of airway safety. As a special type of laryngeal mask, the endoscopic LMA has been shown to be safe and effective in upper gastrointestinal endoscopic surgery [20]. There are also reports on its application in minimally invasive cardiovascular surgery such as atrial fibrillation radiofrequency ablation [21].

Our study also showed that compared with the LMA Supreme group, the blood staining degree of the mask (*P* = 0.04), postoperative sore throat (*P* = 0.03), postoperative oral hemorrhage (*P* = 0.02) in the JEA group were significantly lower (Table 3). According to A. M. Lo’pez’s study, the most frequent postoperative complication was the presence of blood on the LMA at removal, considered slight in all patients, blood was present on LMA Supreme in 7% [11]. Gill RK *et al*. [22] also reported that slight blood staining was present on LMA Supreme (8%). However, Our results showed that rate of blood staining on LMA Supreme reached 17%, which might be related to perioperative anticoagulant therapy, while the rate in JEA group was only 4%, due to their particular material composition and structure (Fig. 1). On one hand, the large-volume inflatable cuff is made of silica gel material, which has high elasticity and flexibility, while the LMA Supreme is a second generation polyvinyl chloride (PVC) single-use device [23, 24]. On the other hand, one of the important features of the LMA Supreme is its reinforced tip and molded distal cuff, designed to prevent posterior folding at insertion [23, 24], while the head end of JEA’s cuff is a semi-open buffer sheet structure, which could minimize the resistance damage to the oropharynx and mucosa around the glottis during the placement process.

According to previous studies, although LMA placement avoids direct irritation and injury to the glottis and trachea, it could cause damage to soft tissue of mouth, pharynx and larynx, sore throat and bleeding were still common postoperative complications [25]. Belena’s study found that during laparoscopic cholecystectomy with LMA Supreme, postoperative sore throat at 2 h was 17% [12], which was lower than our study (18%). Moreover, during cerebral aneurysm embolization with LMA Supreme general anesthesia, we found that patients who received anticoagulant and antiplatelet aggregation therapy experienced significantly increased pharyngeal pain and bleeding after surgery. Therefore, based on the characteristics of surgery for perioperative anticoagulant patients, we need a ventilation device that not only ensures the convenience of implantation, effective ventilation but also reduces oropharyngeal injuries.

There are several limitations in our study. First, and for obvious reasons, the anesthesiologist involved in timing the events in the operating room was not blind to the type of device. To mitigate this, postoperative outcome assessors and patients were blinded to the group assignment. Second, we only investigated the date about the airway management in patients undergoing perioperative anticoagulation for cerebral aneurysm interventional embolization, but we did not record the detailed date on anticoagulation time and degree, which might have resulted in bias and could have affected the power of study. However, preoperative anticoagulation therapy for patients with cerebral aneurysms is consistent with norms and basically unified.

In conclusion, we found that JEA can not only achieve comparable one-time success rate of implantation and quality of ventilation as the LMA Supreme, but also have lower blood staining degree of mask and less sore throat, which confirmed the superiority of JEA as an airway management tool in patients undergoing cerebral aneurysm interventional embolization.

## Data Availability

The datasets generated and/or analysed during the current study are available from the corresponding author upon reasonable request.

## Abbreviations

JEA: Jcerity endoscoper airway
LMA: Laryngeal mask airway
BIS: Bispectral index
FBS: Fiber optic bronchoscope
